# A new species of *Peltidium* Philippi, 1839 (Crustacea, Copepoda, Harpacticoida) from the Pacific coast of Mexico

**DOI:** 10.3897/zookeys.325.5726

**Published:** 2013-08-20

**Authors:** Eduardo Suárez-Morales, Jani Jarquín-González

**Affiliations:** 1 El Colegio de la Frontera Sur (ECOSUR), Unidad Chetumal. A. P. 424, Chetumal, Quintana Roo 77014, Mexico

**Keywords:** Crustacean fauna, marine copepods, phytal meiobenthos, associated copepods, taxonomy

## Abstract

During the analysis of phytal meiobenthic samples collected from a rocky-sandy beach in the state of Nayarit, in the Mexican Pacific, several specimens of harpacticoid copepods were obtained and taxonomically examined. These specimens were found to represent an undescribed species of the peltidiid genus *Peltidium* Philippi, 1839. The new species, *Peltidium nayarit* **sp. n.** is described herein. It resembles *Peltidium nichollsi* Geddes and *Peltidium lerneri* Geddes from Bahamas but also the widespread *Peltidium speciosum* Thompson & Scott and *Peltidium purpureum* Philippi. The new species from the Mexican Pacific differs from its known congeners by its possession of a unique combination of characters, including a modified pectinate seta on the antennary exopod, three terminal setae on the second endopodal segment of leg 1, third exopodal segment of leg 1 with three elements, inner terminal claw twice as long as outer claw, female fifth leg with 5 exopodal setae, exopodal setae I-III stout, spinulose and seta IV being as long as seta V. This is the second species of the family known to be distributed in the Eastern Tropical Pacific and in Mexico. Pending additional data, the distribution of this species appears to be restricted to this area of the Mexican Pacific.

## Introduction

Research on phytal meiobenthos has been advancing in many regions, but there are large areas in which this important community has received little attention (Song et al. 2010). Taxonomic study of these communities is a basic step in monitoring their abundance and diversity patterns. Members of the harpacticoid copepod family Peltidiidae Claus are usually recorded from sandy beaches and live associated with algal patches. They have dorso-ventrally flattened bodies adapted to cope with the strong water flow related to their habitat (Hicks 1986). Currently, this family contains 9 genera (Boxshall and Huys 2013) that represent different lineages of which *Peltidium* Philippi and *Parapeltidium* Scott appear to be closest to the ancestral forms (Hicks 1986). The genus *Peltidium* is the most diverse group among peltidiids. It is known to contain 24 species (Boxshall and Halsey 2004, Wells 2007) but the number of species assigned to this taxon has varied depending on the authors criteria. Nicholls (1941) recognized 19 species, Lang (1948) included 15 and Bodin (1997) recognized 14 species. Wells (2007) recognized that several nominal species that have been assigned to this genus still have an uncertain status.

**Figure 1.**
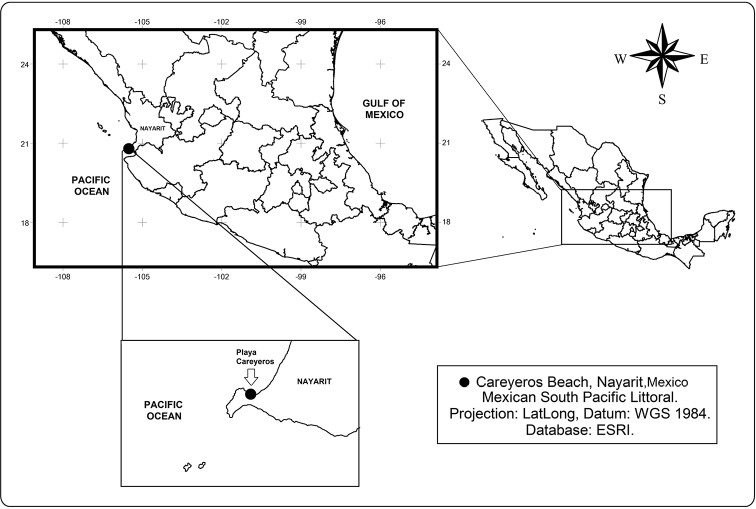
Location of Playa Careyeros, in the State of Nayarit, on the Pacific coast of Mexico, the type locality of *Peltidium nayarit* sp. n.

Playa Careyeros (Fig. 1) is a rocky-sandy area on the southern coast of Nayarit, on the Pacific coast of Mexico. It is influenced by the California Current and the North Equatorial current, with high salinity, temperature gradients and local patterns of coastal circulation (Serviere et al. 1993). During a survey of the local phytal meiofauna, harpacticoid copepods were sorted from samples obtained in algal patches. Several specimens of an harpacticoid copepod that represents a previously undescribed species of the peltidiid genus *Peltidium* Philippi, 1839 were recorded and taxonomically studied. The new species is here described and illustrated; it is compared with its known congeners.

## Methods

A biological survey of the littoral habitats of Playa Careyeros, Nayarit was performed in March 2013. Qualitative samples of algae were taken manually during free diving samplings in the littoral environments of the surveyed area, mainly of algae associated with coral rock at depths not exceeding 3 m. Harpacticoid copepods were extracted by washing the sample through a set of 1.1 and 0.59 mm sieves. Copepods were fixed in 96% ethanol and were then sorted from the original samples and transferred to 70% ethanol with glycerine for long-term preservation. Selected specimens were then placed in glycerol for taxonomical examination and dissection. The dissected appendages were mounted on slides using glycerol as mounting medium and sealed with Entellan®, a fast-drying sealant. Figures were drawn with the aid of a camera lucida. Observations were made with an Olympus BX51 with Nomarski DIC microscope. Morphological terminology follows Huys and Boxshall (1991) and Huys et al. (1996), the systematic arrangement and authority of the family follows Wells (2007). Type specimens were deposited in the Collection of Zooplankton at El Colegio de la Frontera Sur, Chetumal, Mexico (ECO-CHZ) and in the National Museum of Natural History, Smithsonian Institution (NMNH-SI), Washington, D.C.

## Results

### Order Harpacticoida Sars, 1903
Family Peltidiidae Claus, 1860
Subfamily Peltidiinae Claus, 1860
Genus *Peltidium* Philippi, 1839

#### Peltidium nayarit sp. n.

http://zoobank.org/A29D34BE-E213-4840-828D-E39DEEC78E22

http://species-id.net/wiki/Peltidium_nayarit

Figs 2 – 4

##### Type material.

Adult female holotype (ECO-CHZ-08979) partially dissected, mounted on glycerine sealed with Entellan®, Playa Careyeros, Nayarit, Mexico, coll. Jani Jarquín-González, Patricia Salazar and Ramiro Gallardo, March 23, 2013, depth=2–3 m, algal patch from coral rock. Paratypes: three adult females, partially dissected, slides, mounted in glycerine sealed with EntellanÒ, same site, date, and collector (ECO-CHZ-08980), seven undissected adult female specimens preserved in ethanol, vial (ECO-CHZ-08981), same site, date and collector. Two undissected adult females (USNM-1221050), same sampling data.

##### Type locality.

Playa Careyeros (20°46'59.46"N, 105°30'35.48"W), state of Nayarit, central part of the Pacific coast of Mexico.

##### Etymology.

The species is named after the Mexican state of Nayarit, where this species was originally collected. The name of the species is a noun used in apposition.

##### Descriptions.

**Figure 2.**
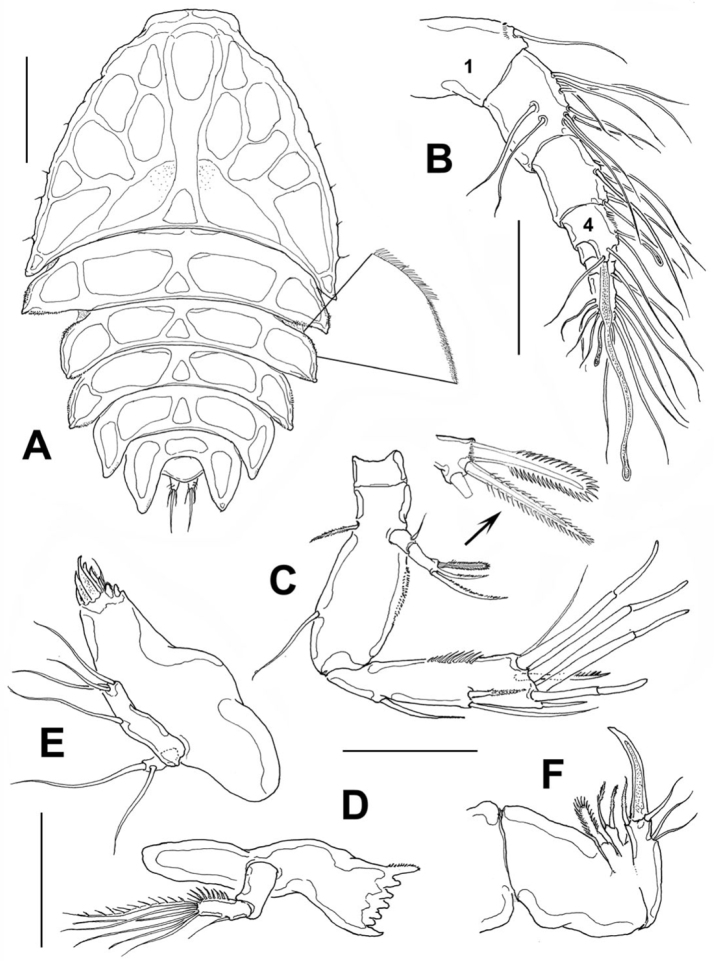
*Peltidium nayarit* sp. n., from Playa Careyeros, Nayarit, Mexican Pacific. **A** adult female, habitus, dorsal view, showing detail of ornamentation of epimeral processes of cephalothorax **B** antennule **C** antenna **D** mandible **E** maxillule **F** maxilla. Scales bars: **A**= 250 μm, **B–F**=100 μm.

*Female*: Body (Fig. 2A) broad, dorsoventrally flattened, arched along longitudinal axis. Cephalosome accounting for about half the body length, with its greatest width at first pedigerous somite, behind which the body gradually tapers posteriorly. Epimera of genital somite and preceding somite pointed and backwardly directed. Length of holotype: 1.03 mm measured from tip of rostrum to posterior margin of anal somite. Length range of type females from 0.93 mm to 1.05 mm, average length 0.96 mm, *n*=16. Rostrum fused to cephalosome, broad, downwardly directed.

**Figure 3.**
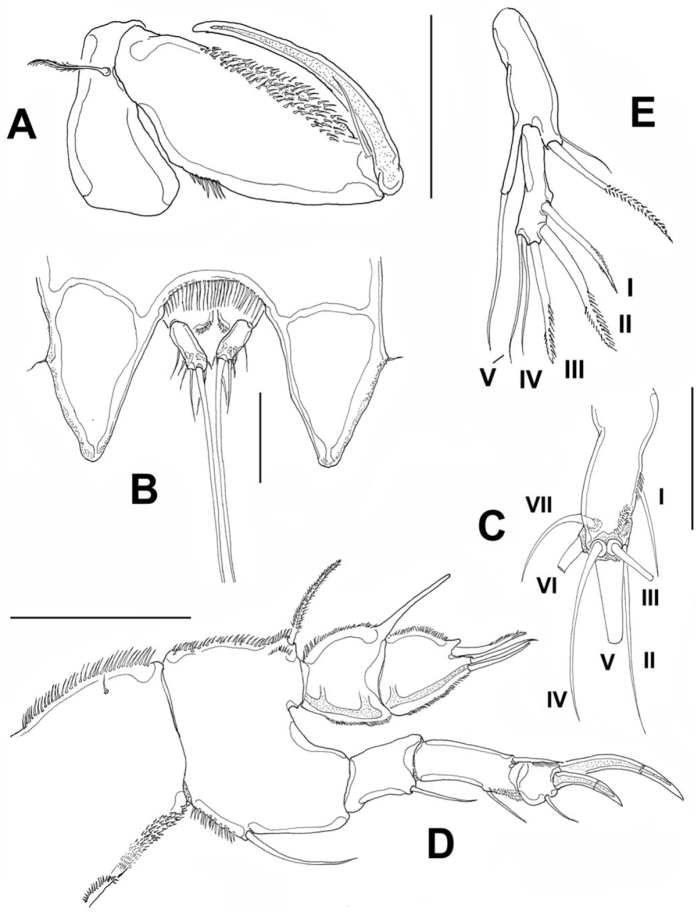
*Peltidium nayarit* sp. n., adult female from Playa Careyeros, Nayarit, Mexican Pacific. **A** maxilliped **B** urosome showing anal somite and caudal rami, ventral view **C** right caudal ramus, ventral view showing setation following nomenclature by Huys et al. (1996) **D** leg 1 **E** leg 5 showing setal nomenclature of exopodal setae following Wells (2007). Scales bars: **A, D, E**=100 μm, **B**= 50 μm, **C**=20 μm.

Cephalosome with a few small, sparsely distributed sensilla on lateral margins (Fig. 2A), posterior margin smooth. Succeeding prosomites, bearing legs 2-4, with flat, laterally expanded subtriangular margins ornamented with a mixed pattern of long and minute spinules (detail in Fig. 2A). First urosomite, bearing leg 5, slightly longer than succeeding genital double-somite; posterior margin of genital double-somite smooth. Anal somite with rounded posterior margin; somite naked in dorsal view, but with a row of long setae along ventral margin (Fig. 3B). Anal area moderately deep, with inner rows of short setules along margin of anal operculum. Caudal rami cylindrical, about twice as long as wide, with 7 setae. Middle caudal apical seta (V) longest. Dorsal seta (VII) about half as long as ramus (Fig. 3C).

Antennule (Fig. 2B) 7-segmented; first segment slightly longer than second, ornamented with distal row of spinules. Armature of antennulary segments (s=setae, ae=aesthetasc) as: 1(1), 2(10s), 3(6s), 4(3s+2ae), 5(1s), 6(2s), 7(9s+1ae). Aesthetasc on fourth segment long, about 70% of antennule length.

Antenna (Fig. 2C). Coxa small, Allobasis with short abexopodal seta and longitudinal patch of spinules on outer margin. Exopod two-segmented, elongated, first segment with short slender seta, second segment bearing three setae distally, distal margin with row of short spinules. One exopodal seta modified, with regular pectinate ornamentation along both margins (see detail in Fig. 2C). Free endopodal segment with outer row of long spinules, armed with one spine and two lateral setae plus seven distal setal elements, four of them being articulated stout setae.

Mandible (Fig. 2D) short, tapering distally. Gnathobasis with narrow diastema, armed with 5-6 monocuspidate teeth, plus stout dorsal seta fused to gnathobasis, ornamented with row of short setules. Mandibular palp small, represented by a subrectangular coxa-basis segment and a single-segmented endopod armed with an inner row of spinules, six terminal setae plus a single outer seta.

Maxillule (Fig. 2E). Praecoxal arthrite armed with eight teeth and one distal short seta. Coxa and basis fused, indistinguishable, with three terminal setae. Endopodite represented by one seta. Exopodite one-segmented, with two setae.

Maxilla (Fig. 2F). Syncoxa robust, short, with two endites, the proximal small and bearing one broad seta, distal endite cylindrical, armed with two subequal, pinnate setae; allobasis forming a strong terminal claw and with one inner basal seta, one outer basal seta and two subequal outer endopodal setae.

Maxilliped (Fig. 3A) subchelate. Coxa and basis elongate. Basis with smooth surface, bearing a well-developed seta, as depicted. First endopodal segment robust, ornamented with single row of spinules and large patch of spinules, as shown. Endopodal claw slender, slightly curved, about 1.3 times as long as basis, with single accompanying seta.

Leg 1 (Fig. 3D). Coxa elongate, ornamented with single row of small spinules on inner and outer margins, plus single short seta on inner middle part of segment. Basis wide, inner margin and part of outer margin ornamented with spinules. Inner distal basipodal seta reaching distal margin of first endopodal segment. Outer basipodal seta reaching distal end of basis. Exopod three-segmented, second exopodal segment longest, about 1.5 times as long as first segment, with patch of minute spinules on outer distal margin. Two exopodal claws on distal position of third exopodal segment; outer claw half as long as inner claw. Endopod two-segmented, shorter than exopod. Endopodal segments wide, globose (sensu Wells 2007), ornamented with rows of short setules along the inner and outer margins. Terminal elements include a spine ornamented with distal row of minute spinules and two equally long slender setae.

**Figure 4.**
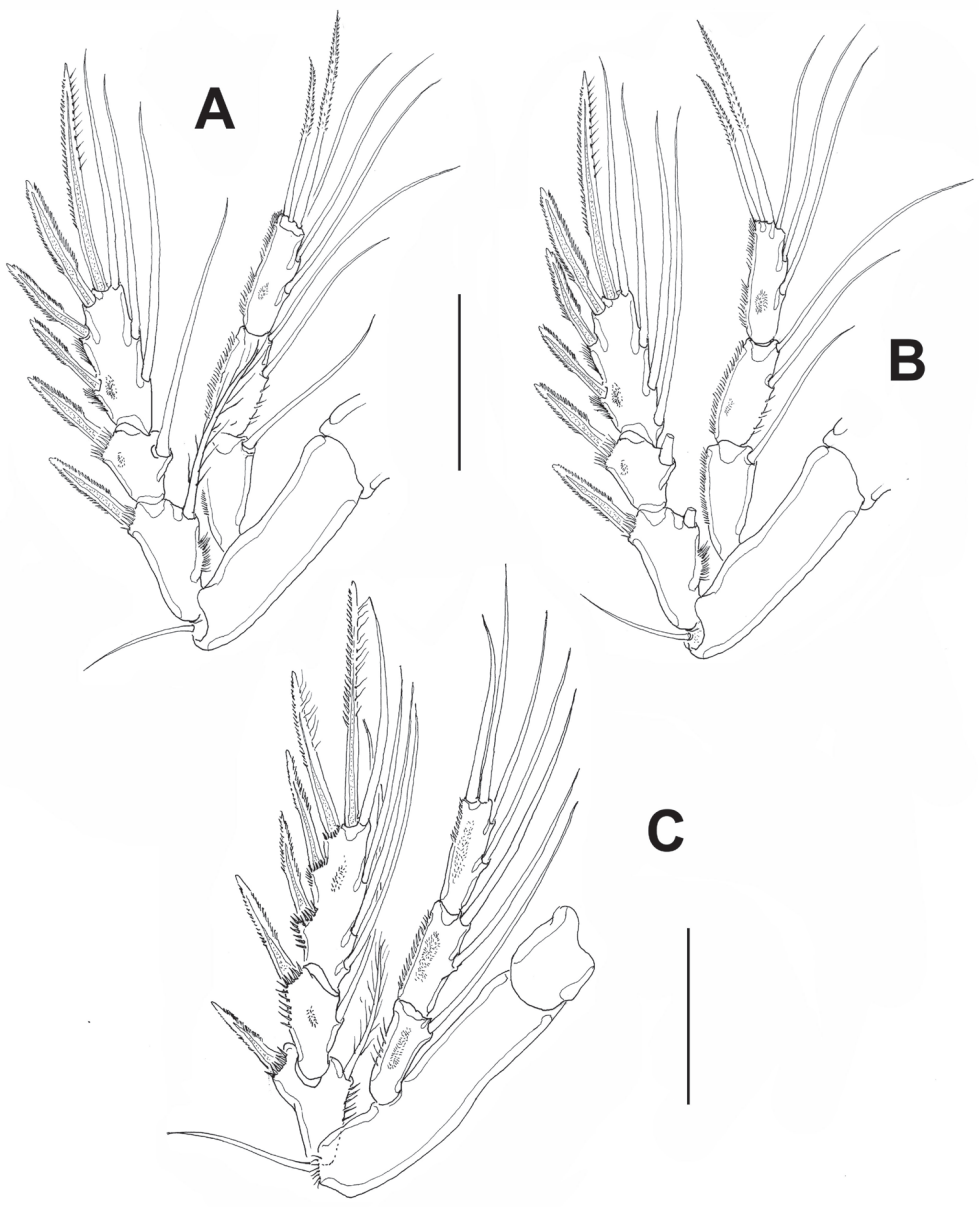
*Peltidium nayarit* sp. n., adult female from Playa Careyeros, Nayarit, Mexican Pacific. **A** leg 2 **B** leg 3 **C** leg 4. Scales bars: **A–C**=100 μm.

Leg 2 (Fig. 4A). Coxa small, basis transversely elongated. Basis with outer seta. Endopod three-segmented, longer than exopod, exopod reaching midlength of third endopodal segment. Exopod three-segmented, with spinules on outer margins of third segment and spinules at insertion of spines on first and second segments.

Leg 3 (Fig. 4B). Coxa, basis, and relative length of endopodal and exopodal rami as in leg 2. Endopod and exopod three-segmented.

Leg 4 (Fig. 4C). Coxa and basis as in leg 3. Exopod three-segmented, third segment with 8 setal elements. Insertion points of exopodal spines and outer margins of second and third exopodal segments with rows of spinules. Endopod three-segmented, slightly longer than exopod, outer margins of segments ornamented with rows of short spinules.

Armature of swimming legs 1-4 as in Table 1.

Leg 5 (Fig. 3E) exopod and baseoendopod separated. Baseoendopod bearing single inner seta. External seta long, borne on elongate cylindrical lobe of baseoendopod reaching half the length of exopodal lobe. Exopodite slender, with 5 setal elements (I-V) (sensu Wells 2007), two inserted on inner margin (I, II), three (III-V) distal. Elements I-III represented by stout, distally pinnate setae, elements IV and V represented by equally long slender setae.

Male: Unknown.

**Table 1.** Armature of swimming legs 1-4 (spines in Roman numerals, setae in Arabic) of *Peltidium nayarit* sp. n. Sequence follows external to internal positions.

	basis	endopod	exopod
leg 1	1-1	0-I; 2,I	1-0;1-1; 1,II
leg 2	1-0	0-1;0-2;II,1,2	I-0;I-1;III,I,1,2
leg 3	1-0	0-1;0-2;II,1,2	I-0;I-1;III,1,2
leg 4	1-0	0-1;0,2;2,2	I-1; I-1; III,I,1,3

##### Remarks.

The available keys to the species of *Peltidium* include those by Nicholls (1941), Lang (1948), and Wells (2007); when following the latter work, our specimens from Nayarit key down to a couplet leading to either *Peltidium nichollsi* Geddes, 1968 or *Peltidium lerneri* Geddes, 1968, both from the Bahamas. These species share the following characters with *Peltidium nayarit* sp. n.: female leg 5 with separate exopod and baseoendopod with 5 setae borne on distal and inner edges only; leg 1 with two-segmented endopod, distal endopodal segment with three setal elements; the number of setae on the distal endopodal segment of legs 2-4 is 3:5:4. The new species differs from these two Bahamian species in several characters, as follows. It diverges from *Peltidium nichollsi* in having a sharper frontal protuberance of the cephalosome and a longer cephalosome (length/width ratio= 1.26 vs. 1.43 in *Peltidium nayarit* sp. n.). In the new species the outer basipodal seta of leg 1 is slender, inserted on the proximal half of segment, whereas this seta is stouter, shorter, and borne on the distal half of the basipod in *Peltidium nichollsi*. The setal elements of both the exopod and endopod of leg 1 differ in these two species. The third exopodal segment *Peltidium nichollsi* bears two strong, stout subdistal elements in addition to the pair of terminal claws (Geddes 1968, fig. 6B) vs. a single slender seta in *Peltidium nayarit* sp. n. (Fig. 3D) and the terminal claws are equally long in *Peltidium nichollsi*, whereas the inner claw is about twice as long as the outer one in the new species. In *Peltidium nichollsi* the three distal elements of the second endopodal segment have different lengths, whereas these elements are equally long in *Peltidium nayarit* sp. n. Both species share the presence of one modified seta on the antennary exopod, but in *Peltidium nichollsi* only the tip of the seta is irregularly pectinate (Geddes 1968, fig. 7B), whereas in the new species this seta is regularly pectinate almost its entire length (see detail in Fig. 2C). Another species with a distally pectinate exopodal seta is *Peltidium maldivianum* Sewell, 1940, but it differs in characters of leg 1 and leg 5 from both *Peltidium nichollsi* (see Wells 2007) and *Peltidium nayarit* sp. n. The armature of leg 5 is also different between the new species and *Peltidium nichollsi*. In the latter, setae I and II are heavily pectinate (Geddes 1968, fig. 7G), they are also clearly shorter than setal elements III and V, and seta IV is shorter than elements III and V. In the new species elements I-III are not heavily pectinate but lightly spinulose (Fig. 3E), element II is longest and seta IV is almost as long as seta V.

The new species can be distinguished from *Peltidium lerneri* by the armature of leg 1. In *Peltidium lerneri* the exopodal claws are subequally long, whereas the inner is twice as long as the outer one in *Peltidium nayarit* sp. n. In addition, the inner spiniform seta of the second endopodal segment is clearly shorter than the other two setae *vs*. an equal length of the three elements in the new species. Also, as indicated by Wells (2007), in *Peltidium lerneri* the shape of the first endopodal segment is quadrate (Geddes 1968, fig. 8C), with straight outer margin and inner margin only slightly convex; this character allows distinction from the globose (i.e. both margins of segment convex) condition present in both *Peltidium nichollsi* and *Peltidium nayarit* sp. n. The middle of the three antennary exopodal setae is the longest in *Peltidium lerneri* (Geddes 1968, fig. 9B), but the corresponding seta is the shortest in the new species (Fig. 2C). Most importantly, the female leg 5 is different in these two species. In *Peltidium lerneri* setal element IV is very reduced, about 1/3 as long as adjacent seta V (Geddes 1968, fig. 8G), whereas the same element is as long as seta V in the new species (Fig. 3E). A reduced seta IV is also present in *Peltidium proximum* Nicholls, 1941 (Nicholls 1941). Also, the exopodal lobe is clearly shorter in *Peltidium lerneri* (length/width ratio=1.8) (Geddes 1968, fig. 8G) than it is in *Peltidium nayarit* sp. n., which is an elongate, slender structure (L/W ratio=4.14).

The new species shares also some important characters with *Peltidium speciosum* Thompson & Scott, 1903 (see Nicholls 1941), including a leg 5 with a very similar armature and structure except for a relatively robust exopodal segment (length/width ratio= 3.7 vs. 4.2 in *Peltidium nayarit* sp. n.) and a shorter outer baseoendopodal seta, reaching to about half the length of exopodal seta V (Nicholls 1941, fig. 8), thus differing from the new species, in which the same seta almost reaches the distal end of seta V (Fig. 3E). In the new species one of the antennary exopodal setae is modified, as described (Fig. 2C), whereas *Peltidium speciosum* lacks modified setae (Nicholls 1941 fig. 8). Several characters of leg 1 differ in these two species; in *Peltidium speciosum*, the inner basipodal seta is relatively longer than in the new species, it reaches to about half the length of the second endopodal segment (Nicholls 1941, fig. 8), whereas it is clearly shorter in *Peltidium nayarit* sp. n., barely reaching halfway along the first endopodal segment. The shape of the first and second endopodal segments differs in these two species, the first one is subquadrate as in *Peltidium lerneri* (Nicholls 1941, Geddes 1968) and the second is subrectangular, with straight margins, thus diverging from the subtriangular shape present in *Peltidium nayarit* sp. n. (Fig. 3D). Most importantly, in *Peltidium speciosum* there are four setal elements on the second endopodal segment, thus diverging from the new species (and also from *Peltidium lerneri* and *Peltidium nichollsi*), with only three such elements. The terminal exopodal claws of leg 1 are subequal in *Peltidium speciosum* and diverge from the pattern described herein for *Peltidium nayarit* sp. n. When Nicholls’ (1941) work is followed to identify our specimens from Nayarit, we reach *Peltidium purpureum*, which can be easily separated from the new species by its having 4 setal elements (2 claws, 2 setae) on the third exopodal segment of leg 1 vs. 3 elements (2 claws, 1 seta) in the new species, a different shape of the endopodal segments of leg 1, with narrower segments, and by its having 6 setal elements on the exopodal lobe of leg 5 instead of five found in *Peltidium nayarit* sp. n. Also, *Peltidium purpureum* has 3 maxillar endites (1 proximal, 2 distal) while *Peltidium nayarit* and all other *Peltidium* species mentioned in this paper (except maybe *Peltidium maldivianum*, whose maxilla has not been described) have lost the proximal endite.

## Discussion

*Peltidium* is a very widely distributed genus with records from different regions of the world but it is not very diverse in a given area, for instance only three species are known from the Mediterranean: *Peltidium gracile* (Claus, 1889), *Peltidium purpureum* Philippi, 1839, and *Peltidium robustum* (Claus, 1889) (Todaro and Cecherelli 2010), two species are known to occur in East Asia (Song and Yun 1999), and only five species of *Peltidium* have been known to occur in the Caribbean region (Varela 2005, Suárez-Morales et al. 2006). Records include the recently described *Peltidium proximus* Varela, 2005 from Cuba, which should not be confused with Nicholls’ (1941) *Peltidium proximum* from South Australia. The Eastern Pacific region is not an exception, only a few species of Peltidiidae have been recorded and among them there are only some unidentified records of *Peltidium* (Lang 1965, Cordell 2006). Hitherto, there were no previous records of Peltidiidae in Mexican waters of the Atlantic and the Pacific oceans (Suárez-Morales et al. 2000). Recently, Gómez and Varela (2013) described a new species of the peltidiid genus *Alteutha* Baird, 1846 from Sinaloa, southern part of the Gulf of California, northwest Mexico. The finding of the new species *Peltidium nayarit* represents the first species of *Peltidium* described for Mexico and the second record of Peltidiidae known in this country. Because of the scarce taxonomical surveys of the phytal meiofauna in the Eastern Tropical Pacific, and the record of only one species of *Peltidium* so far, it is assumed that the family and genus diversity remains underestimated in the region.

## Supplementary Material

XML Treatment for Peltidium nayarit
